# Living on the edge: Daily, seasonal and annual body temperature patterns of Arabian oryx in Saudi Arabia

**DOI:** 10.1371/journal.pone.0180269

**Published:** 2017-08-30

**Authors:** S. Streicher, H. Lutermann, N. C. Bennett, M. F. Bertelsen, O. B. Mohammed, P. R. Manger, M. Scantlebury, K. Ismael, A. N. Alagaili

**Affiliations:** 1 Department of Zoology and Entomology, University of Pretoria, Pretoria, South Africa; 2 KSU Mammals Research Chair, Department of Zoology, College of Science, King Saud University, Riyadh, Saudi Arabia; 3 Copenhagen Zoo, Centre for Zoo and Wild Animal Health, Frederiksberg, Denmark; 4 School of Anatomical Sciences, Faculty of Health Sciences, University of the Witwatersrand, Johannesburg, South Africa; 5 School of Biological Sciences, Institute for Global Food Security, Queen's University Belfast, Belfast, Northern Ireland, United Kingdom; 6 Prince Saud Al- Faisal Wildlife Research Centre, Taif, Saudi Arabia; 7 Saudi Wildlife Authority, Riyadh, Saudi Arabia; State Museum of Natural History, GERMANY

## Abstract

Heterothermy, the ability to allow body temperature (T_b_) to fluctuate, has been proposed as an adaptive mechanism that enables large ungulates to cope with the high environmental temperatures and lack of free water experienced in arid environments. By storing heat during the daytime and dissipating it during the night, arid-adapted ungulates may reduce evaporative water loss and conserve water. Adaptive heterothermy in large ungulates should be particularly pronounced in hot environments with severely limited access to free water. In the current study we investigated the effects of environmental temperature (ambient, T_a_ and soil, T_s_) and water stress on the T_b_ of wild, free-ranging Arabian oryx (*Oryx leucoryx*) in two different sites in Saudi Arabia, Mahazat as-Sayd (MS) and Uruq Bani Ma’arid (UBM). Using implanted data loggers wet took continuous T_b_ readings every 10 minutes for an entire calendar year and determined the T_b_ amplitude as well as the heterothermy index (HI). Both differed significantly between sites but contrary to our expectations they were greater in MS despite its lower environmental temperatures and higher rainfall. This may be partially attributable to a higher activity in an unfamiliar environment for translocated animals in UBM. As expected T_b_ amplitude and HI were greatest during summer. Only minor sex differences were apparent that may be attributable to sex-specific investment into reproduction (e.g. male-male competition) during rut. Our results suggest that the degree of heterothermy is not only driven by extrinsic factors (e.g. environmental temperatures and water availability), but may also be affected by intrinsic factors (e.g. sex and/or behaviour).

## Introduction

Deserts sustain life, despite being hot and dry with low and unpredictable precipitation, intense solar radiation, low primary production and soil fertility, extreme span of ambient temperatures as well as an overall lack of free standing water [1–3]. Water availability is likely to be the most important factor affecting live in deserts and primary productivity often closely parallels rainfall in desert ecosystems [4]. Similarly, since free standing water is usually scant in deserts, secondary productivity is usually indirectly linked to water availability as the rate of food consumption by desert herbivores tends to be linked to the water content of the plants consumed [5–8]. As a result animals living in desert environments make use of behavioural and physiological adaptations to reduce water loss and conserve energy [9,10]. Among mammals behavioural responses in the form of seasonal migrations, such as the large scale migrations of wildebeest (*Connochaetus taurinus*) in East Africa and springbok (*Antidorcas marsupialis*) in southern Africa but also the more localized movements of gemsbok (*Oryx gazelle*) in the Namib, are well documented [9]. Migration is, however, usually restricted to lager mammals in which locomotion is not to costly and the use of retreats can provide short-term relief [9]. Consequently, many desert-dwelling mammals exhibit nocturnal activity and use burrows during the day [9,11]. Their body size prevents large mammals from the use of the latter strategy. Instead shade seeking behaviour is commonly observed while in the absence of shade ungulates have also been observed to orientate themselves in a way that the least body surface is exposed to the sun [9,12,13]. Although their larger body size is associated with a reduction in evaporative water loss (EWL), the more limited benefits of the behavioural strategies employed, probably means that they have a to rely on physiological mechanisms to a greater extent for water and energy conservation than small mammals [9].

Endotherms generally regulate and maintain their core body temperatures (T_b_) within a narrow range, and when inhabiting deserts, large mammals such as ungulates have developed unique adaptations for thermoregulation [14–17]. Selective brain cooling and adaptive heterothermy are among the main strategies employed by large ungulates [9,13–15,18]. Adaptive heterothermy is a strategy whereby an endotherm relaxes its strict T_b_ limits and allows the T_b_ to increase during the day. Subsequently, these animals may lose stored heat by non-evaporative means during the night allowing them to reduce EWL [19].

Both energy constraints as well as water shortage may induce heterothermy in large mammals [18]. Although both of these factors (i.e. energy and water) may induce heterothermy the physiological mechanism and the resulting T_b_ patterns may differ substantially. Energetic bottlenecks as a result of food shortage and/or higher energetic demands due to cooler climatic conditions can be expected to lead to lower minimum and mean T_b_ (i.e. hypothermia) as a result of a reduction in metabolic rate (MR) in response to low food availability. Consequently, this type of heterothermy is more likely to occur in winter. Even if ambient conditions and energy supply are comparable between the sexes higher reproductive investment of one sex may result in sex-specific heterothermy as has been shown for male camels where males exhibited lower minimum T_b_ than females during rut when males reduce their foraging time in favour of energetically costly vigilance, territoriality and fighting [20]. In contrast, if ambient temperature exceeds T_b_ and low water availability is low the competing demands of body fluid and thermal homeostasis make a reduction in EWL by increasing maximum T_b_ (hyperthermy) a more sustainable strategy for large mammals [10,18].

Heterothermy has been studied in a number of large ungulates in captivity, but studies on free-living ungulates, where the movement of animals have not been restricted, have only been conducted on two species of desert ungulates, the Arabian oryx (*Oryx leucoryx)* and the Arabian sand gazelle (*Gazella subgutturosa marica*) [15,21–23]. The former is the only species of oryx occurring outside of Africa [24]. Once widespread throughout the Arabian Peninsula, the Arabian oryx was extirpated from the wild in 1972 [15,25]. Saudi Arabia currently hosts the two largest free-ranging Arabian oryx populations one in Mahazat as-Sayd, ca. 200 km north-east of Taif, and one in Uruq Bany Ma’arid, in the western Rub-al Khali, one of the driest regions of the world both of which lack permanent water bodies [26–29]. The Arabian oryx is a highly mobile species that can survive months without access to free standing water [15,24]. As a predominantly grazing species they obtain most of their water from several grass species but may supplement their diet by browsing and digging for underground tubers, particularly in summer, due to the deterioration of nutritional value and water content of their main food species [7,8,30–33]. Considered an exploratory species Arabian oryx exhibit highly flexible home ranges with large distance migration being observed after rainfall (mostly during spring in Saudi Arabia) while it will contract again during hot and dry periods (e.g. summer) when shade seeking appears to govern movement patterns [8,15,31,33]. In addition, ranging patterns and feeding strategies may differ between the sexes with older, dominant males becoming territorial at greater population sizes and may change over time since the release in a new habitat [8,30,31,34]. As additional behavioural adaptation to the desert climate Arabian oryx exhibit a switch from diurnal to nocturnal activity in response to high ambient temperatures and water shortage and spend substantial amounts of the daytime in the shade of Acacia trees or grass tussocks where they may dig shallow depressions to facilitate heat exchange [8,31,35–37]. Rainfall has also been shown to affect reproduction in Arabian oryx and although birth may occur throughout the year, both in captivity and in the wild, conception is more likely to occur after rainfall [8,29,31,37,38].

Like most artiodactyls, Arabian oryx have been found to employ selective brain cooling in response to water limitations [39]. However, compared with other arid-adapted ungulates including the closely related gemsbok, Arabian oryx exhibited a lower threshold and increased frequency of selective brain cooling while the magnitude was similar [39,40]. The authors found furthermore some evidence that the use of this water conservation mechanism may be modified by behaviour and one dominant male in their study did employ brain cooling to a lesser degree and at higher thresholds [39].

Both in captivity und in free-ranging populations Arabian oryx respond with T_b_ reduction to food restriction while maintaining a high digestive efficiency [6,7,41]. In addition, Arabian oryx show a remarkably low EWL when food and water restricted and have one of the lowest water influx rates reported for desert ungulates including camels [7,41]. However, these may be increased in lactating females due to their higher water requirements [7].

The T_b_ in the Arabian oryx has previously been studied in one of the free-ranging Saudi Arabian populations Both studies found that Arabian oryx employ adaptive heterothermy with maximum T_b_ amplitudes in summer of 4.1 and 7.7°C, respectively, presumably as a strategy to reduce water loss and energy costs [15,21]. Although both the highest and the lowest T_b_ were recorded during summer (hot-dry season) the seasonal patterns of heterothermy were largely attributed to water restrictions [21]. Both of these studies had relatively low sample sizes and/or were only conducted during restricted periods in Mahazat as-Sayd and comparative data from other localities experiencing a different climate as well as data allowing investigating possible sex-specific patterns are currently lacking.. Consequently, in the current study we examined T_b_ of free-ranging male and female Arabian oryx at two different localities in Saudi Arabia that differ greatly in the mean annual rainfall receive. We hypothesized that (1) we would observe that Arabian oryx employ adaptive heterothermy. Furthermore, we predicted that (2) the frequency and amplitude of heterothermy would be larger in the more arid site and (3) during summer when high temperatures coincide with low precipitation. Lastly, we anticipated to observe sex differences in heterothermy patterns coinciding with reproductive activity (i.e. rut and the calving season)

## Material and methods

### Study areas

#### Mahazat as-Sayd

Mahazat as-Sayd (MS) is a fenced protected area located in west-central Saudi Arabia (22°15’ N, 41°40’E), comprised of 2553 km^2^ of open steppe desert. The substrate composition at MS is loose sand, gravel and alluvial clays [42]. The mean minimum and maximum temperatures recorded between 1991 and 2009 were 9°C and 42°C respectively [27]. Characteristic of an arid climate, MS experiences hot summers and mild winters with very low rainfall [15]. Data for the past 18 years show great inter-annual variation in rainfall, with an average of ±7 mm rainfall per month from 1991 to 2009 [27]. Vegetation is sparse, predominated consisting of perennial grasses with sporadically distributed small *Acacia* trees providing shade for animals [43].

MS was the first release site for the reintroduction of Arabian oryx in Saudi Arabia [26,28]. Between 1990 and 1994 a total of 76 captive-bred were released in MS. These included individuals from the breeding programme at the National Wildlife Research Center (NWRC) in Taif (derived from the donation of 57 animals from the farm of late King Khaled in Thumamah) and supplemented by captive bred animals from the World Heard (comprising of descendants of founders from Aden Protectorate, Kuwait, Saudi Arabia as well as wild-caught individuals from London Zoo), Jordan, Bahrain and Qatar [26,28,29]. The population size now exceeds 300 individuals and based on management recommendations for this population, animals above an estimated carrying capacity of 70% are regularly removed and either incorporated in the NWRC breeding programme or relocated to other suitable habitats such as Uruq Bani Ma’arid. Since the first reintroduction in 1995 from the NWRC breeding herd into Uruq Bani Ma’arid it has grown to one of more than 200 individuals [26,28].

#### Uruq Bani Ma’arid

Uruq Bani Ma’arid (UBM) is Saudi Arabia’s largest unfenced protected area and covers a surface area of approximately 12787 km^2^ [42]. It is located at the western edge of the Empty Quarter, 200 km north of Najran in southern Saudi Arabia, (19.10°N, 45.3°E) [42]. UBM is characterized by a hyper-arid climate, very low to sporadic rainfall (less than 25 mm per annum), with rainfall absent for a few years, extremely high ambient temperatures and very low floristic diversity, providing little to no shade for animals [44,45]. Annual ambient temperature and average monthly rainfall from 1985 to 2009 were 28.4°C and 3.2 mm respectively [27]. Accordingly, UBM is one of the driest and harshest regions in the world [26,42,46].

We obtained measurements of ambient (T_a_) and soil temperature (T_s_) routinely recorded by the reserve staff in MS and UBM every 15 min throughout the year at weather stations located within the respective reserves (MS: Rumrumiyyah, UBM: Sharurah). Daily rainfall measurments made at the same weather stations were also obtained.

### Anaesthesia and logger implantation

Twenty animals (8 males, 12 females) were captured in MS and after implantation, half of the animals were released back into MS (4 males, 6 females) and the other half was released at UBM (4 males, 6 females). All animals were remote injected using a Dan-Inject dart gun (Daninject, Børkop, Denmark) with etorphine hydrochloride (CaptivonTM 98, Wildlife Pharmaceuticals Ltd., White River, South Africa; 19μg/kg; a semi-synthetic opioid that has an analgesic effect and is an opioid receptor antagonist), Ketamine (Ketaminol^®^ Vet., MDS Animal Health, Intervet International B.V., Boxmeer, The Netherlands; 0.3mg/kg; a dissociative anaesthetic agent which is an NMDA receptor antagonist), Midazolam (Midazolam, Wildlife Pharmaceuticals Ltd., White River, South Africa; 0.13mg/kg; a benzodiazepine class anaesthetic agent that acts by enhancing the effect of GABA on GABAA receptors), and Medetomidine (Zalopine 10 mg/ml, Orion Pharma, Espoo, Finland; 5μg/kg; a sedative analgesic that is an alpha-2 adrenergic agonist). All animals received oxytetracycline (Terramycin LA, Pfizer, Brazil, 20mg/kg IM; a broad spectrum antibiotic) and ketoprofen (Ketovet, Vetmedim Animal Health, Cantho City, Vietnam, 2mg/kg IM; a proprionic acid class of nonsteroidal anti-inflammatory which acts to inhibit the production of prostaglandin). After the operations, anaesthesia was reversed using naltrexone hydrochloride (Naltrexone, APL, Kungens Kurva, Sweden; 40mg IM; an opiate antagonist) and atipamezole hydrochloride (Antisedan, Orion Pharma; 2mg IM; a synthetic 2 adrenergic receptor antagonist). Using aseptic techniques, a data logger that measured temperature was inserted intra-abdominally (AIC001: Abdominal implant, Africa Wildlife Tracking, South Africa). The T_b_ device was calibrated against an accurate thermometer in an insulated water bath, providing an accuracy of better than 0.06°C. A small incision (less than 80 mm in length) was made through the skin at the implantation site and the muscle layers of the abdominal wall were separated using blunt dissection. The implant, pre-coated in inert wax and sterilized within a container with formalin pellets for 48 hr prior to implantation, was inserted untethered into the abdomen, the incisions closed, and liberally sprayed with Necrospray. A satellite collar with a GPS unit was attached to the neck of each animal (iridium satellite collar, Africa Wildlife Tracking, South Africa, Model: SBU202). Following reversal of anaesthesia, the animals were monitored in holding pens with access to food and water for 10 days, before being released into the two protected areas for the year-long recording period. The animals were not recaptured following the recording period, the collars and implants being left *in situ*.

### Body temperature measurement

The T_b_ of Arabian oryx were monitored in both habitats and the precise location of each oryx were traced using iridium satellite collars with connections to a body temperature logger (http://www.atstrack.com). In standard operation the tracking device will upload the location of the collar and temperature of the implant to the database immediately after position acquisition. Thus, data were made available immediately eliminating the wait for bulk uploads. Uploads for the T_b_ occurred every 10 min throughout the day and night. The on-board temperature sensor made measurements of T_b_ to an accuracy of 0.5 of a °C for 6 equal intervals each and every hour.

### Statistical analysis

The daily variance in T_b_ was calculated for each animal in MS and UBM using two different methods. First, we calculated the daily temperature amplitude by subtracting the minimum from the maximum T_b_ for each animal every 24 hours [15,22]. As a second index of daily variance in T_b_ we quantified the variation in T_b_ using the Heterothermy Index (HI) of Boyles *et al*. [47]:

$$HI=\sqrt{\frac{\sum {\left(T_{b-\text{mod}}-T_{b-i}\right)}^{2}}{n-1}}$$

where T_b-mod_ is the modal T_b_, T_b-i_ is the T_b_ measurement at time *i* and *n* is the number of times T_b_ is sampled. The HI quantifies deviation away from the theoretically optimal temperature for performance as approximated by T_b-mod_. T_b-mod_ was calculated as the modal T_b_ for individuals that displayed unimodal distributions of T_b_ and the mode of the highest peak for individuals that displayed bimodal distributions of T_b_ [48,49] HI values were calculated for each animal over each 24 h period [47].

We compared the body mass between male and female Arabian oryx released in MS and UBM, respectively, using a general linear model (GLM). We included sex and site (MS or UBM) as well as the interaction between sex and site as independent variables in the model.

We explored the fluctuations of mean hourly T_a_ and T_s_ using linear mixed models (LMMs). We included site (MS, UBM), hour and season (winter: December-February, spring: March-May, summer: June-August and autumn: September-November, [21]) as well as all 2- and 3-way interactions between these variables in our model.

To evaluate the possible contributions of study site, season and an animal’s sex on mean daily T_b_ amplitude and HI, respectively, we employed linear mixed models (LMMs). All 2- and 3-way interactions between these variables were included in the model. Oryx ID was included as random effect in our models to account for the repeated measurement of the same individual. These analyses were carried out using the ‘lme’ function from the ‘nlme’ package in R using restricted maximum likelihood. Non-significant terms were dropped sequentially from the model based on the ΔAIC [50] and only the results of the final model are reported.

To determine the relationship between T_b_ and extrinsic factors, we tested for a correlation between hourly average T_b_ and T_a_ and T_s_ for both sites over the entire study period as well as for each season in each site. All statistical tests were completed using the software package *R* (version 2.15.3). All data are presented as mean ± SD and p<0.05 was considered significant.

Ethical clearance for the current study was obtained from the University of the Witwatersrand Animal Ethics Committee (clearance certificate number 2014/53/D) and all animals were treated according to the guidelines of this committee, which parallel those of the National Institute of Health of South Africa (NIH) for the care and use of animals in scientific studies. Permission to work in MS and UBM was granted by the President of the Saudi Wildlife Authority (SWA).

## Results

### Climate

During the study period the average annual T_a_ in MS was 26.85 ± 8.15°C and the T_s_swas 30.44 ± 9.54°C. The T_a_ fluctuated during the four seasons and was lowest in winter (14.93 ± 5.44°C) and highest in summer (33.05 ± 4.91°C, Table 1). The T_s_ was consistently higher than the air temperature (Table 1).

**Table 1:** The mean T_b_, T_a_, T_s_, and rainfall in MS and UBM during winter (Dec—Feb), spring (Mar—May), summer (Jun—Aug), autumn (Sep—Nov) and for the whole year (March 2014 to February 2015).

	Annual	Winter	Spring	Summer	Autumn
	mean ± SD	mean ± SD	mean ± SD	mean ± SD	mean ± SD
**T**_**b**_ **MS (n = 8)**	38.23 ± 0.80°C	38.20 ± 0.50°C	38.29 ± 0.81°C	38.35 ± 1.00°C	38.01 ± 0.81°C
T_a_	26.85 ± 8.15°C	14.93 ± 5.44°C	23.52 ± 8.88°C	33.05 ± 4.91°C	26.07 ± 6.41°C
T_s_	30.44 ± 9.54°C	16.77 ± 5.91°C	26.74 ± 10.34°C	37.64 ± 6.81°C	29.49 ± 7.59°C
Rainfall	28. 81 mm	0.34 mm	112.1 mm	0 mm	3.22 mm
**T**_**b**_ **UBM (n = 6)**	38.26 ± 0.77°C	38.00 ± 0.47°C	38.43 ± 0.47°C	38.43 ± 0.84°C	38.14 ± 0.65°C
T_a_	30.32 ± 8.60°C	18.25 ± 5.22°C	27.72 ± 5.17°C	33.9 ± 4.21°C	26.39 ± 5.80°C
T_s_	33.81 ± 8.06°C	20.9 ± 4.78°C	31.31 ± 6.35°C	37.39 ± 5.81°C	30.32 ± 6.33°C
Rainfall	0.87 mm	0 mm	3.14 mm	0 mm	0 mm

In UBM, the average yearly T_s_ was 33.81 ± 8.06°C and the T_a_was 30.32 ± 8.60°C. The T_a_ varied during the four seasons and was lowest in winter (18.25 ± 5.22°C) and highest in summer (33.9 ± 4.21°C, Table 1). Just as in MS, the T_s_ was always higher than the air temperature in UBM (Table 1).

**Fig 1:**
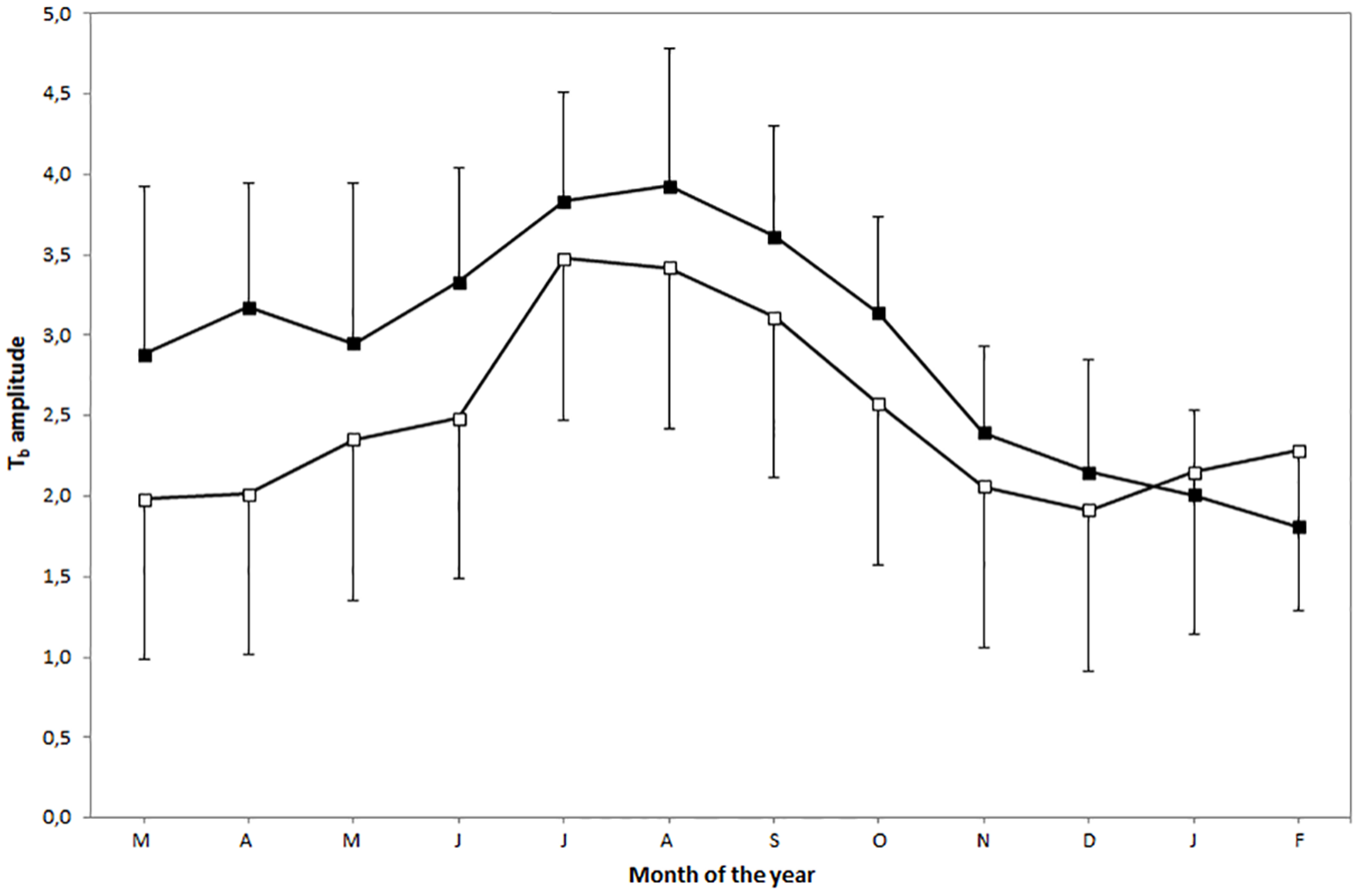
The mean monthly T_b_ amplitude (± SD) for oryx in MS (filled squares) and UBM (open squares) from March 2014 to February 2015.

The mean hourly T_a_ did not differ significantly between the two study sites (df = 1, 15874; F = 0.18; p = 0.67). At the same time, T_a_ differed significantly between the seasons (df = 3, 334; F = 340.04; p < 0.0001) and during the day (df = 1, 15874; F = 1101.57; p < 0.0001, Fig 1). The interaction between site and season was significant (df = 3, 15874; F = 53.34; p < 0.0001, Fig 1). Similarly, the interaction between site and hour was significant (df = 1, 15874; F = 8.392; p = 0.0038, Fig 1). In contrast, the interaction between season and hour was not significant (df = 3, 15874; F = 1.61; p = 0. 19). The 3-way interaction between site, season and hour was significant (df = 3, 15874; F = 3.43; p = 0.0164, Fig 1).

The mean hourly T_s_ did not differ significantly between the study sites (df = 1, 15874; F = 1.90; p = 0.17). In contrast, it differed significantly between the seasons (df = 3, 334; F = 470.96; p < 0.0001) and hours (df = 1, 15874; F = 2375.30; p < 0.0001). In addition, the interaction between site and season was significant (df = 3, 15874; F = 15.92; p < 0.0001). The T_s_ did not differ significantly with hours between seasons (df = 1, 15874; F = 3.52; p = 0.061). Conversely, the interaction between season and hour was significant (df = 1, 15874; F = 6.56; p = 0.0002). Similarly, the 3-way interaction between site, season and hour was significant (df = 3, 15874; F = 3.65; p = 0.0120).

Rainfall was low in MS throughout the year, but summer was the only season during which no precipitation was recorded. In contrast, the only rainfall recorded for UBM occurred in spring which also coincided with the greatest mean precipitation in MS (Table 1).

### Body mass of Arabian oryx

The mean body mass of oryx released in the MS was 70.5 ± 13.7 kg, n = 10 range 48–88 kg (males 77.6 ± 12 kg, n = 4; range 54–88 kg and females 63 ± 11.4 kg, n = 6, range 48–78 kg) while it was 67.0 ± 13.1 kg, n = 10 range 48–90 kg (males 69.8 ± 2.4 kg, n = 4; range 68–74 kg and females 64.2 ± 20 kg, n = 6, range 48–90 kg) for animals released in UBM. The body mass of our study animals did neither differ significantly between sites (F = 1.44, df = 1, p = 0.248) nor between the sexes (F = 4.11, df = 1, p = 0.060). Similarly, the interaction between site and sex was not significant (F = 1.24, df = 1, p = 0.281). Due to the remote nature of the current study no additional body mass measurements could be taken during the course of the study.

### Mean T_b_

Several study animals were lost due to poaching and in other instances the transmitters failed. Of the released animals T_b_ data could be retrieved from 8 (3 males, 5 females) and six (2 males, 4 females). For these oryx we recovered T_b_ data for a mean of 318.07 ± 20.87 days (range: 99–365). The mean yearly T_b_ for 8 individuals in MS was 38.24 ± 0.93°C. It varied between seasons and ranged from 38.20 ± 0.50°C in winter to 38.35 ± 1.00°C in summer (Table 1). In UBM the mean yearly T_b_ for 6 animals was 38.26 ± 0.77°C. The mean T_b_ ranged from 38.00 ± 0.47°C in winter to 38.43 ± 0.84°C in summer (Table 1). We found a significant effect of season on average T_b_ in UBM (ANOVA: df = 3, F = 8.47, p<0.01).

There were no seasonal differences between MS and UBM, except during winter (Mann-Whitney U test: W = 3179.5, p = 0.02). The average T_b_ was lower in UBM (38.00 ± 0.47°C) when compared with MS (38.20 ± 0.50°C).

### Daily T_b_ variation

**Fig 2:**
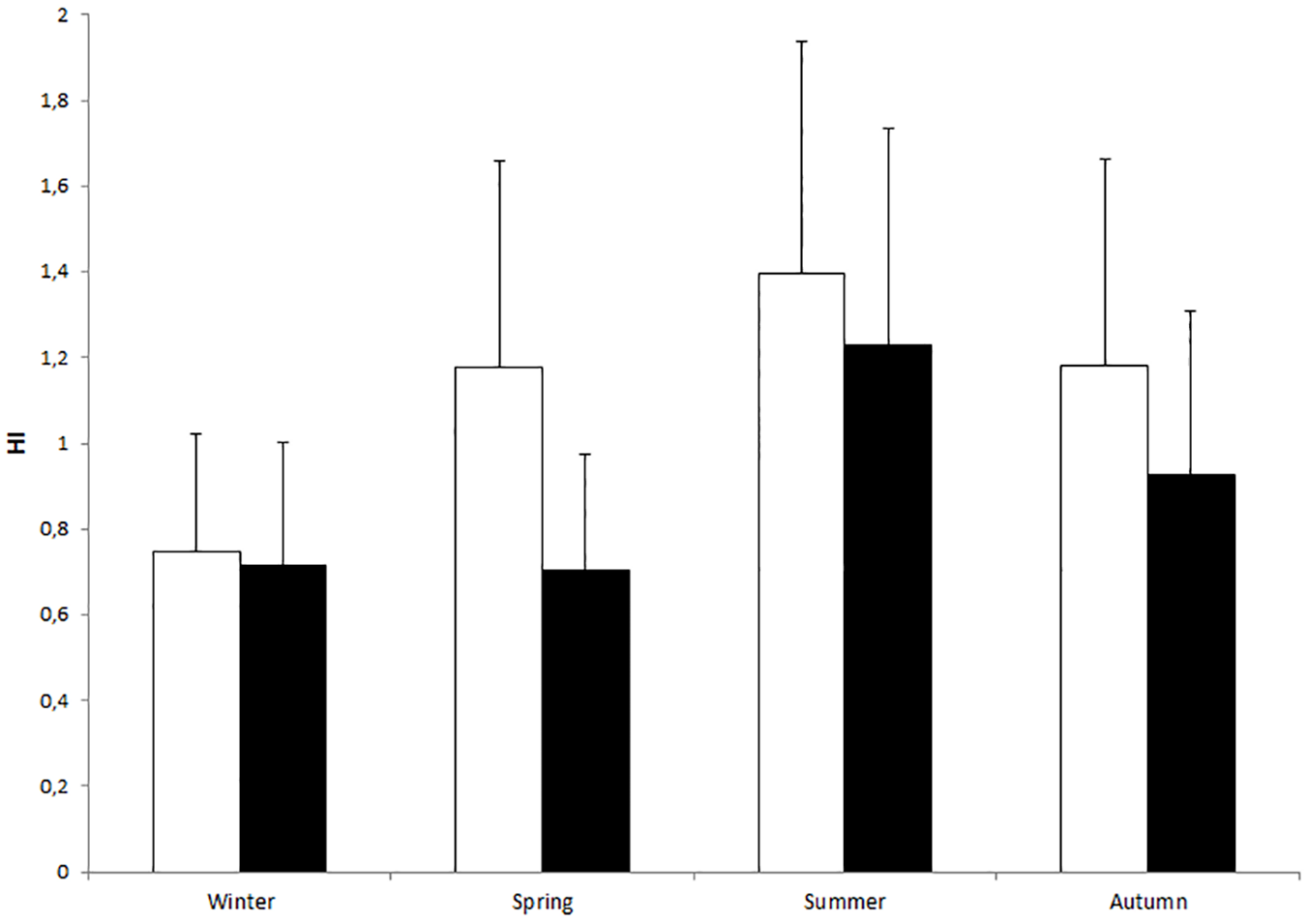
Traces of T_b_ of an oryx for five consecutive days during February (grey line, representing winter) and five consecutive days during August (black line, representing summer) from (a) MS and (b) UBM.

The LMM indicated that the mean T_b_ amplitude was significantly greater in MS (2.97 ± 0.98°C) compared with UBM (2.53 ± 0.83°C; df = 1,11; F = 8.44; p = 0.0143). It also differed significantly between seasons (df = 3,4468; F = 736.65; p < 0.0001, Fig 1). Oryx had the lowest mean daily T_b_ amplitude during winter (2.05 ± 0.62°C) and the highest during summer (3.41 ± 0.82°C, Fig 2). Mean T_b_ amplitude did not differ significantly between the sexes (df = 1,11; F = 4,57; p = 0.0559). The interaction between site and season was significant (df = 3,4468; F = 61.95; p < 0.0001). The mean T_b_ amplitude was lower in UBM than in MS in spring but not the other seasons. In addition, the interaction between season and sex was significant (df = 3,4468; F = 6.48; p = 0.0002) with males, but not females exhibiting a significantly larger amplitude in summer compared with winter.

### Heterothermy index

**Fig 3:**
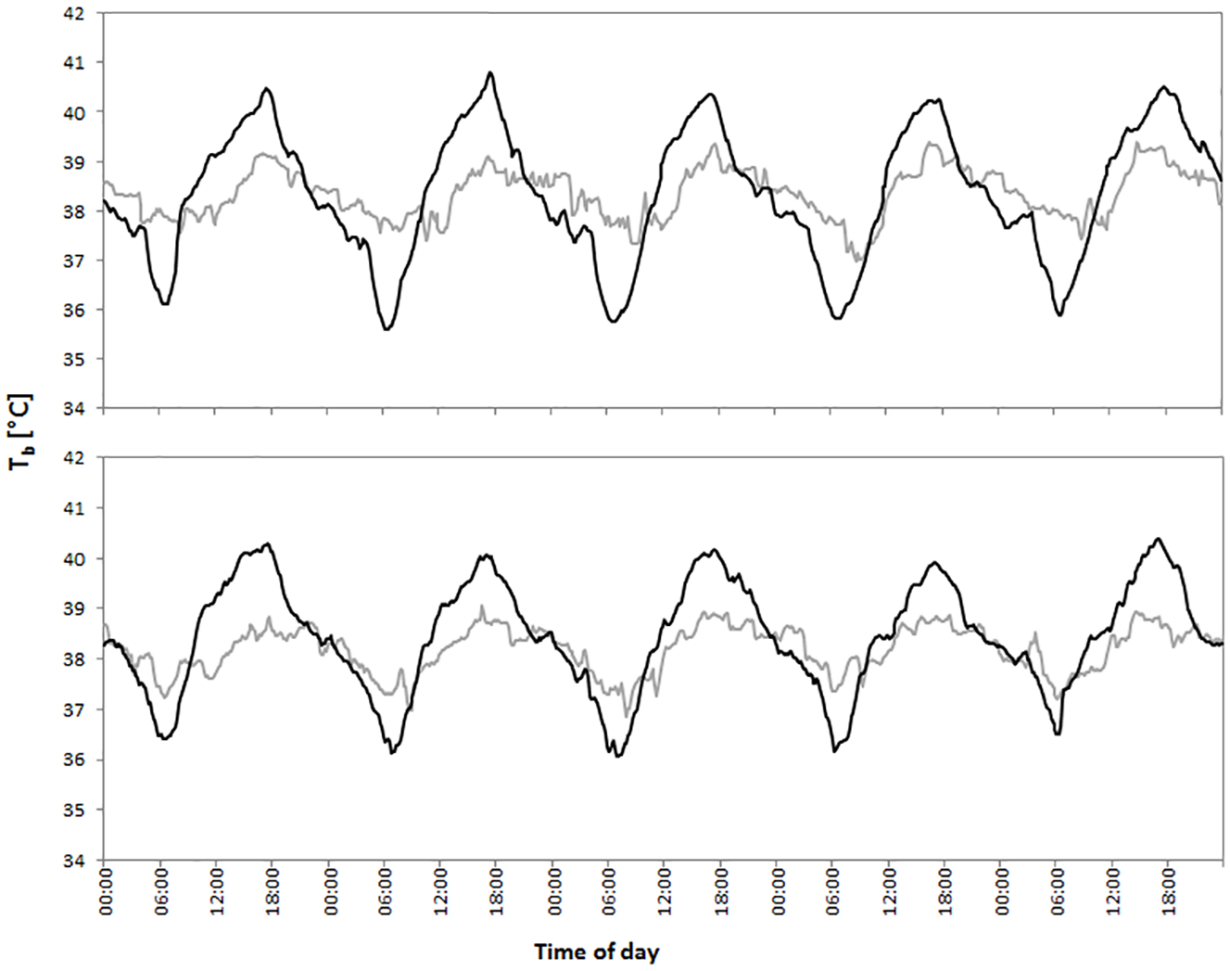
Seasonal variation in HI (± SD) in MS (open bars) and UBM (closed bars).

The HI was significantly greater in MS (1. 56 ± 0.53) compared with UBM (0.91 ± 0.44; df = 1,11; F = 13.17; p = 0.0040). In addition, the HI varied significantly between seasons and was greatest in summer (df = 3,4430; F = 427.47; p < 0.0001, Table 1). However, HI did not differ significantly between the sexes (df = 3,4430; F = 3.71; p = 0.080). The interaction between site and season was significant (df = 3,4430; F = 38.56; p < 0.0001) and in spring but not the other seasons HI was greater in MS than UBM (Fig 3). Similarly, the interaction between season and sex was significant (df = 3,4430; F = 4.66; p = 0.0030) with male but not female HI being significantly greater in summer compared with winter.

### Correlation between T_a_, T_s_ and T_b_

**Fig 4:**
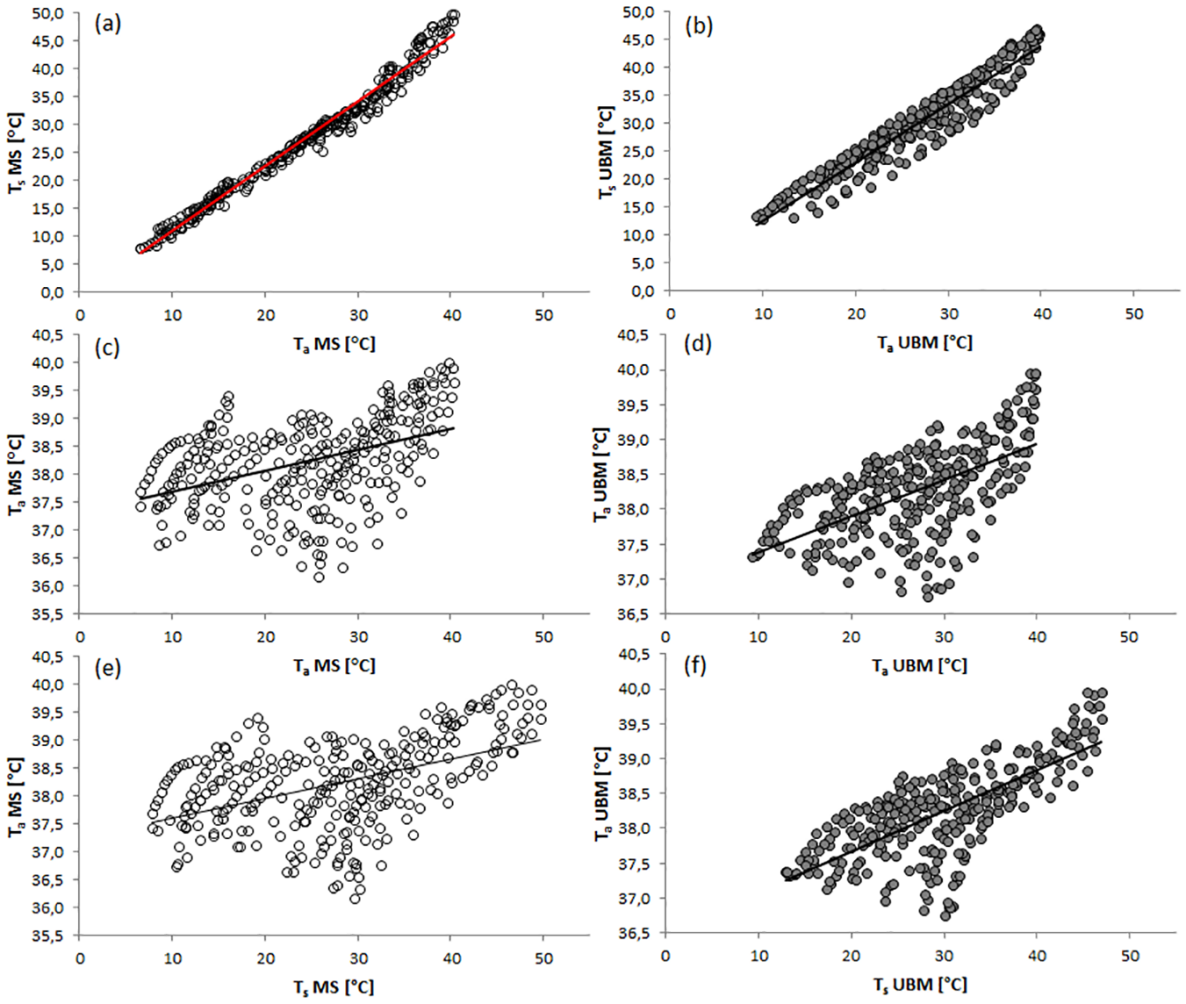
Correlation between (a) T_a_ and T_s_ as well as T_a_ and T_b_ and T_s_ and T_b_ in MS (a,c, e) and UBM (b, d, f).

Throughout the year in both MS (Spearman: S = 24076.07, rho = 0.99, p<0.01) and UBM (Spearman: S = 173116, rho = 0.96, p<0.01) there was a positive correlation between *T*_*a*_ and *T*_*s*_ (Fig 4). In addition, *T*_*a*_ and T_b_ (Spearman: S = 2141865, rho = 0.46, p<0.01) as well as *T*_*s*_ and T_b_ (Spearman: S = 2058695, rho = 0.49, p<0.01) were significantly correlated in MS (Fig 4c and 4e).

**Fig 5:**
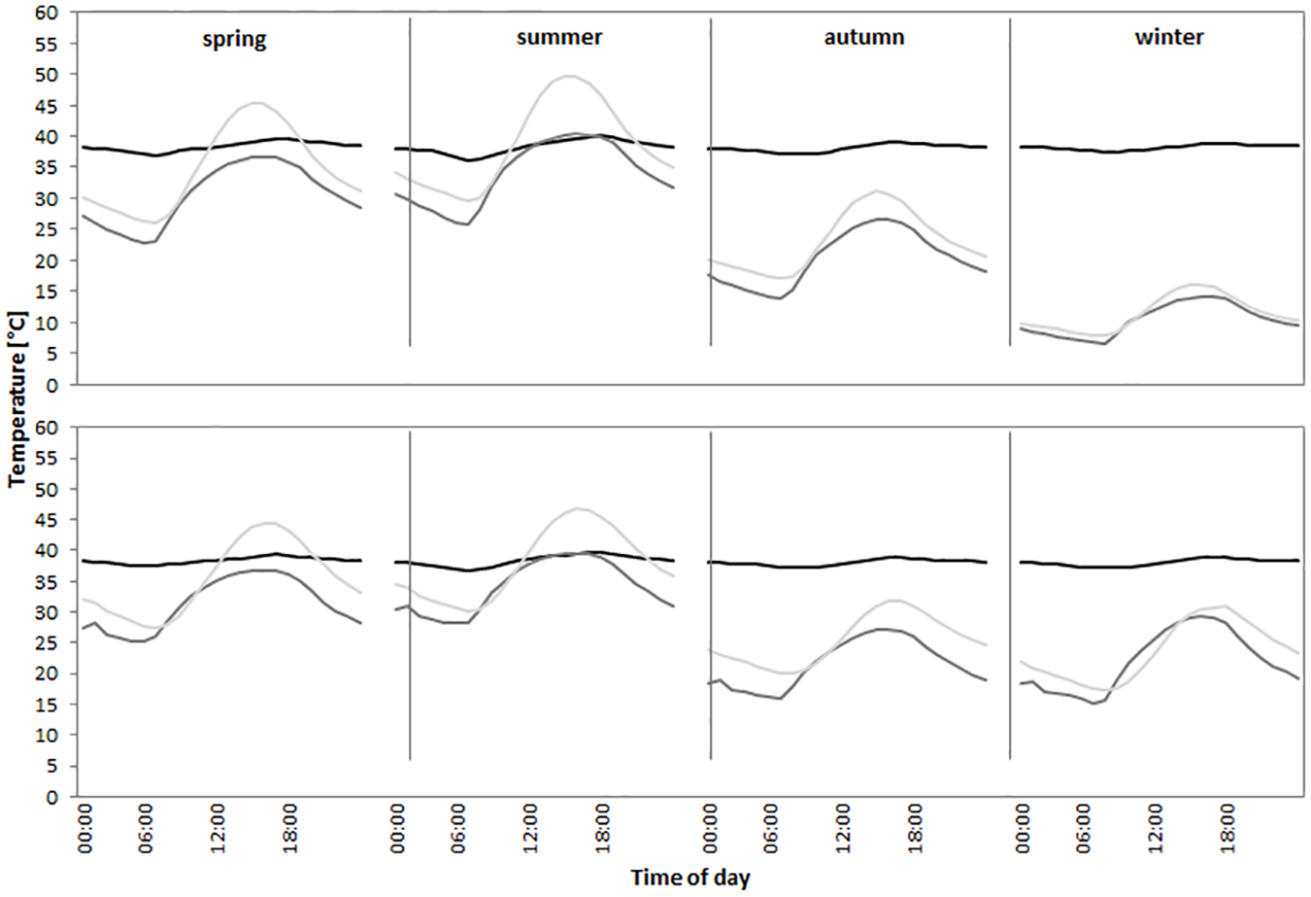
Correlation between the seasonal variation of mean hourly T_b_ (black line), T_a_ (dark grey line) and T_s_ (light grey line). Displayed are temperature traces for MS (top panel) and UBM (bottom panel).

In MS, when we compared hourly average T_b_ to *T*_*a*_ and *T*_*s*_ for every season, we detected a significant correlation for winter (*T*_*a*_: Spearman: S = 30030.64, rho = 0.52, p<0.01; *T*_*s*_: Spearman: S = 31306.02, rho = 0.50, p<0.01), spring (*T*_*a*_: Spearman: S = 28224.58, rho = 0.55, p<0.01; *T*_*s*_: Spearman: S = 27674.56, rho = 0.56, p<0.01) and autumn (*T*_*a*_: Pearson: t = 5.41, df = 70, rho = 0.54, p<0.01; *T*_*s*_: Pearson: t = 6.67, df = 70, rho = 0.62, p<0.01) and summer (*T*_*a*_: Spearman: S = 10230.73, rho = 0.84, p<0.01; *T*_*s*_: Spearman: S = 8221.86, rho = 0.87, p<0.01) (Fig 5). We found a significant correlation between *T*_*a*_ and *T*_*s*_ for all four seasons (Fig 5). Similarly, T_b_ increased significantly with increasing *T*_*a*_ in UBM (Spearman: S = 1612055, rho = 0.60, p<0.01, Fig 4b) for the whole year. Likewise, T_b_ was significantly correlated with *T*_*s*_ (Spearman: S = 1106835, rho = 0.72, p<0.01, Fig 4d and 4e).

In UBM, when we looking at hourly average *T*_*b*_, *T*_*a*_ and *T*_*s*_ for every season, we detected significant correlations for all seasons: winter (*T*_*a*_: Spearman: S = 23792.68, rho = 0.62, p<0.01; *T*_*s*_: Spearman: S = 8144.92, rho = 0.87, p<0.01), spring (*T*_*a*_: Spearman: S = 25066.93, rho = 0.60, p<0.01; *T*_*s*_: Spearman: S = 14057.42, rho = 0.77, p<0.01) and autumn (*T*_*a*_: Spearman: S = 25552.23, rho = 0.59, p<0.01; *T*_*s*_: Spearman: S = 12291.59, rho = 0.80, p<0.01) and summer(*T*_*a*_: Spearman: S = 9433.18, rho = 0.85, p<0.01; *T*_*s*_: Spearman: S = 3246.75, rho = 0.95, p<0.01, Fig 5). We found a significant positive correlation between *T*_*a*_ and *T*_*s*_ for all four seasons (Fig 5).

## Discussion

Our study provides the first continuous body temperature measurements for an entire calendar of free-living Arabian oryx in UBM within the ‘Empty Quarter’ in Saudi Arabia. Our data show that, as has previously been reported for MS [21], oryx in UBM display the characteristics typically associated with adaptive heterothermy—an increase in the daily amplitude of T_b_ rhythm where maximum T_b_ increased and minimum T_b_ decreased, particularly during the dry and hot months (i.e. summer). As predicted the two methods we used to quantify body temperature fluctuations, T_b_ amplitude and HI, both confirmed that oryx in UBM employ adaptive heterothermy. The use of HI, suggested to provide and objective reference for heterothermy measurements, has been criticized because of its reliance on the upper mode of the bimodal temperature distribution typical for small mammals that may not necessarily reflect the optimal temperature and does not account for variation in MR [18,47,51,52]. However, although the inclusion of simultaneous MR and T_b_ measurements has been suggested to provide a more accurate indication of the use of heterothermy this is logistically challenging in studies like ours [53]. The similar results obtained for T_b_ amplitude and HI furthermore suggest that the disadvantages of the latter are negliable in our study.

Our study provides the first comparison of T_b_ patterns between Arabian oryx from two different areas in Saudi Arabia, MS (a rocky desert) and UBM (a sandy desert). However, contrary to our expectations both T_b_ amplitude and HI were significantly greater in the less water restricted habitat (i.e. MS). At the same time, the annual average T_b_ for six oryx in UBM was 38.26 ± 0.77°C and comparable to the T_b_ in MS and what has previously been reported from the latter site (38.24 ± 0.93°C) for which water has been suggested as important cue for adaptive heterothermy during periods of water restriction [15,18,21]. This suggests that, contrary to what has been hypothesized [21], water shortage is unlikely to be the key driver of heterothermy in our study animals. Similarly, based on their observations in Arabian sand gazelles Ostrowski and Williams [22] stated that heterothermy is not a response to water stress in desert-adapted ungulates. The significance of water restriction as driver for heterothermy in ungulates has furthermore been questioned by Grigg et al. [20] who observed greater heterothermy in rutting camel males during winter when water was freely available. Also, our data confirm that water is substantially more restricted in UBM than in MS throughout the year with rainfall only recorded during spring in UBM while it was absent only during summer in MS. At the same time, while T_a_ and T_S_ in summer are similar in both sites they are substantially lower during winter and spring in MS compared with UBM. Alternatively, animals in UBM may have exerted a stronger control over their T_b_ by employing behavioural mechanisms, such as seeking out shade or shifting their activity period to the cooler periods of the day, to a greater extent than oryx in MS [15,23,35,36]. Indeed, a heavy reliance on acacia trees for shade on the escarpment in UBM that supersedes foraging requirements has been reported for Arabian oryx in UBM [8]. At the same time studies in UBM as well as of other oryx populations suggest that Arabian oryx released in a new environment range over much larger distances than those already familiar with their environment [8,30,31,33]. In addition, territorial aggression expressed by dominant males [34] may have forced the newly introduced individuals to exhibit greater activity. In contrast, the individuals re-released in MS were already familiar with the habitat in MS may have quickly resumed their previous home ranges and hence not have experienced changes in their activity patterns. Thus, the additional body heat generated by a greater general activity may at least partially account for the observed difference in heterothermy patterns.

As predicted both the amplitude of T_b_ and the HI varied significantly between seasons and was highest during summer and lowest during winter for both sites. This is likely to be linked to the combined stress of food limitation and water shortage during the hot, dry summer while in winter the cooler temperatures meant that food shortage alone was probably responsible for the observed pattern [6,7,41]. However, although slightly lower than previously reported [15,21] both T_b_ amplitude and HI were significantly greater in MS compared with UBM This result was unexpected given the harsher environmental conditions faced by oryx in UBM and further refutes the hypothesis that adaptive heterothermy is linked to water limitations or high ambient temperatures [21,22].This is further supported by the fact that (almost) no rainfall was recorded during both winter and summer while T_a_ and T_s_ and were substantially higher during the latter season in both sites.

In accordance with our predictions we only found a significant interaction between season and sex with males, but not females exhibiting larger amplitudes of T_b_ and HI in summer, when environmental temperatures peaked, compared with winter with precipitation being low during both of these seasons. Since the sexes did not differ in their body mass we can largely exclude body mass as a contributing factor to this pattern [9,18,23]. Rather, the different reproductive investment between the sexes may have contributed to the observed sex-effects on oryx T_b_ amplitude and HI in our study. Although Arabian oryx in Saudi Arabia may have offspring at any time of the year reproduction is closely tied with rainfall and birth peaks occur in spring [8,29,37]. The species exhibits a postpartum oestrus and the gestation length is roughly 9 months suggesting that rutting mostly occurs in summer [38]. Hence, similar to what has been reported for camels, the additional nutritional stress caused by a reduction in foraging activity in favour of energetic investment in territorial behaviour and fighting among males may account for the observed larger change in our heterothermy measures in males from summer to winter [8,20,34].

Our data suggest that as previously reported for populations in MS Arabian oryx in UBM employ adaptive heterothermy, a process which reduces evaporation by storing body heat during the day, as a strategy to cope with their harsh habitat. However, despite the lower temperatures and greater water availability indices of heterothermy were greater in the cooler, more humid site suggesting that behavioural factors may override the expression of heterothermy in the study species. At the same time the greater variance in T_b_ during the hottest time of the year supports the role of both energy and water limitations on the expression of heterothermy in ungulates. In addition, seasonal variation in sex-specific patterns of heterothermy furthermore suggest that sex differences in reproductive investment may modify the expression of heterothermy in our study animals. Thus, results suggest that the degree of heterothermy was not only driven by extrinsic factors such as environmental temperatures and rainfall but may also be affected by intrinsic factors such as sex and behaviour.

## References

[1] De Vos A. Some observations on ecological adaptations of desert rodents and suggestions for further research work In: Prakash I, Ghosh PK, editors. Rodents in desert environments. Springer Netherlands; 1975 pp. 70–121.

[2] Degen AA. Ecophysiology of small desert mammals. Berlin: Springer Verlag; 1997.

[3] Schmidt-Nielsen K. Animal Physiology: adaptation and environment. Cambridge, UK: Cambridge University Press; 1997.

[4] Noy-Meier I. Desert ecosystems: environment and producers. Annu Rev Ecol Syst. 1973;4: 25–51.

[5] Macfarlane WV, Howard B. Comparative water and energy economy of wild and domestic animals. Symp Zool Soc London. 1792;31: 261–296.

[6] Williams JB, Ostrowski S, Bedin E, Ismail K. Seasonal variation in energy expenditure, water flux and food consumption of Arabian Oryx *Oryx leucoryx*. J Exp Biol. 2001;204: 2301–2311. 11507113 10.1242/jeb.204.13.2301

[7] Ostrowski S, Williams JB, Bedin E, Ismail K. Water influx and food consumption of free-living oryxes (*Oryx leucoryx*) in the Arabian desert in summer. J Mammal. 2002;83: 665–673. doi: 10.1644/1545-1542(2002)083<0665:WIAFCO>2.0.CO;2

[8] Strauss WM. An ecological study of reintroduced Arabian oryx in the ‘Uruq Bani Ma’arid protected area of the Kingdom of Saudi Arabia. University of Pretoria. 2003.

[9] Louw GN, Seely MK. Ecology of desert organisms. New York: Longman Group Ltd.; 1982.

[10] Cain JW, Krausman PR, Rosenstock SS, Turner JC. Mechanisms of thermoregulation and water balance in desert ungulates. Wildl Soc B. 2006;34: 570–581.

[11] Scantlebury M, Danek-Gontard M, Bateman PW, Bennett NC, Manjerovic B, Joubert KE, et al Seasonal patterns of body temperature daily rhythms in group-living Cape ground squirrels *Xerus inauris*. PLoS One. 2012;7: e36053 doi: 10.1371/journal.pone.0036053 22558324 10.1371/journal.pone.0036053 PMC3338621

[12] Lease HM, Murray IW, Fuller A, Hetem RS. Black wildebeest seek shade less and use solar orientation behavior more than do blue wildebeest. J Therm Biol. 2014;45: 150–156. doi: 10.1016/j.jtherbio.2014.08.008 25436964 10.1016/j.jtherbio.2014.08.008

[13] Fuller A, Hetem RS, Maloney SK, Mitchell D, Fuller A, Hetem RS, et al Adaptation to heat and water shortage in large, arid-zone mammals. Physiology. 2014;29: 159–167. doi: 10.1152/physiol.00049.2013 24789980 10.1152/physiol.00049.2013

[14] Mitchell D, Maloney SK, Jessen C, Laburn HP, Kamerman PR, Mitchell G, et al Adaptive heterothermy and selective brain cooling in arid-zone mammals. Comp Biochem Physiol. 2002;131: 571–585. 10.1016/s1096-4959(02)00012-x 11923074

[15] Ostrowski S, Williams JB, Ismael K. Heterothermy and the water economy of free-living Arabian oryx (*Oryx leucoryx*). J Exp Biol. 2003;206: 1471–1478. 12654886 10.1242/jeb.00275

[16] Kinahan AA, Pimm SL, van Aarde RJ. Ambient temperature as a determinant of landscape use in the savanna elephant, L*oxodonta africana*. J Therm Biol. 2007;32: 47–58.

[17] Park N, Rica C, Carrillo E, Saenz JC, Fuller TK. Movements and activities of white-lipped peccaries in Corcovado. New York 2002;108: 317–324.

[18] Hetem RS, Maloney SK, Fuller A, Mitchell D. Heterothermy in large mammals: Inevitable or implemented? Biol Rev. 2016;91: 187–205. doi: 10.1111/brv.12166 25522232 10.1111/brv.12166

[19] Schmidt-Nielsen K, Schmidt-Nielsen B, Jarnum SA, Houpt TR. Body temperature of the camel and its relation to water economy. Am J Physiol. 1956;188: 103–112. 10.1152/ajplegacy.1956.188.1.103 13402948

[20] Grigg G, Beard L, Dorges B, Heucke J, Coventry J, Coppock A, et al Strategic (adaptive) hypothermia in bull dromedary camels during rut; could it increase reproductive success? Biol Lett. 2009;5: 853–856. doi: 10.1098/rsbl.2009.0450 19605390 10.1098/rsbl.2009.0450 PMC2827997

[21] Hetem RS, Strauss WM, Fick LG, Maloney SK, Meyer LCR, Shobrak M, et al Variation in the daily rhythm of body temperature of free-living Arabian oryx (*Oryx leucoryx*): Does water limitation drive heterothermy? J Comp Physiol Bl. 2010;180: 1111–1119. doi: 10.1007/s00360-010-0480-z 20502901 10.1007/s00360-010-0480-z

[22] Ostrowski S, Williams JB. Heterothermy of free-living Arabian sand gazelles (*Gazella subgutturosa marica*) in a desert environment. J Exp Biol. 2006;209: 1421–1429. doi: 10.1242/jeb.02151 16574802 10.1242/jeb.02151

[23] Hetem RS, Strauss WM, Fick LG, Maloney SK, Meyer LCR, Shobrak M, et al Does size matter? Comparison of body temperature and activity of free-living Arabian oryx (*Oryx leucoryx*) and the smaller Arabian sand gazelle (*Gazella subgutturosa marica*) in the Saudi desert. J Comp Physiol B. 2012;182: 437–449. doi: 10.1007/s00360-011-0620-0 22001971 10.1007/s00360-011-0620-0

[24] Stewart DRM. The Arabian oryx (*Oryx leucoryx pallas*). Afr J Ecol. 1963;1: 103–117.

[25] Henderson DS. Were they the last Arabian oryx? Rev Res Arid Zo Hydrol. 1974;12: 347–350.

[26] Mésochina P, Bedin O, Ostrowski S. Reintroducing antelopes into arid areas: lessons learnt from the oryx in Saudi Arabia. C R Biol. 2003;326: 158–165. 10.1016/s1631-0691(03)00053-2 14558465

[27] Islam MZU, Ismail K, Boug A. Restoration of the endangered Arabian Oryx *Oryx leucoryx*, Pallas 1766 in Saudi Arabia lessons learnt from the twenty years of re-introduction in arid fenced and unfenced protected areas: (Mammalia: Artiodactyla). Zool Middle East. 2011;54: 125–140.

[28] Ostrowski S, Bedin E, Lenain DM, Abuzinada AH. Ten years of oryx conservation breeding in Saudi Arabia—achievements and regional perspectives. Oryx. 1998;32: 209–222.

[29] Wronski T, Lerp H, Ismail K. Reproductive biology and life history traits of Arabian oryx (*Oryx leucoryx*) founder females reintroduced to Mahazat as-Sayd, Saudi Arabia. Mamm Biol. 2011;76: 506–511. doi: 10.1016/j.mambio.2011.01.004

[30] Corp N, Spalton A, Gorman ML. The influence of rainfall on range in a female desert ungulate: the Arabian oryx (*Oryx leucoryx*) in the Sultanate of Oman. J Zool. 1998;246: 369–377. doi: 10.1111/j.1469-7998.1998.tb00169.x

[31] Stanley Price MR. Animal reintroductions: the Arabian oryx in Oman. Cambridge, UK: Cambridge University Press; 1989.

[32] Asmodé J-F. Food choice and digging behaviour of naive arabian oryx reintroduced in their natural environment. Rev d’Ecologie—La terre la viecologie. 1990;45: 295–301.

[33] Tear TH, Mosley JC, Ables ED. Landscape-scale foraging decisions by reintroduced Arabian oryx. J Wildl Manage. 1997;61: 1142–1153.

[34] Tear TH, Ables ED. Social system development and variability in a reintroduced Arabian oryx population. Biol Conserv. 1999;89: 199–207. doi: 10.1016/S0006-3207(98)00136-0

[35] Davimes JG, Alagaili AN, Gravett N, Bertelsen MF, Mohammed OB, Ismail K, et al Arabian oryx (*Oryx leucoryx*) respond to increased ambient temperatures with a seasonal shift in the timing of their daily inactivity patterns. J Biol Rhythms. 2016;31: 365–374. doi: 10.1177/0748730416645729 27154303 10.1177/0748730416645729

[36] Hetem RS, Strauss WM, Fick LG, Maloney SK, Meyer LCR, Shobrak M, et al Activity re-assignment and microclimate selection of free-living Arabian oryx: Responses that could minimise the effects of climate change on homeostasis? Zoology. Elsevier GmbH.; 2012;115: 411–416. doi: 10.1016/j.zool.2012.04.005 23036437 10.1016/j.zool.2012.04.005

[37] Ismail K, Kamal K, Plath M, Wronski T. Effects of an exceptional drought on daily activity patterns, reproductive behaviour, and reproductive success of reintroduced Arabian oryx (*Oryx leucoryx*). J Arid Environ. Elsevier Ltd; 2011;75: 125–131. doi: 10.1016/j.jaridenv.2010.09.017

[38] Sempere AJ, Ancrenaz M, Delhomme A, Greth A, Blanvillain C. Length of estrous cycle and gestation in the Arabian oryx (*Oryx leucoryx*) and the importance of the male presence for induction of postpartum estrus. Gen Comp Endocrinol. 1996;101: 235–241. doi: 10.1006/gcen.1996.0026 8729933 10.1006/gcen.1996.0026

[39] Hetem RS, Strauss WM, Fick LG, Maloney SK, Meyer LCR, Fuller A, et al Selective brain cooling in Arabian oryx (*Oryx leucoryx*): a physiological mechanism for coping with aridity? J Exp Biol. 2012;215: 3917–3924. doi: 10.1242/jeb.074666 22899527 10.1242/jeb.074666

[40] Maloney SK, Fuller A, Mitchell G, Mitchell D. Brain and arterial blood temperatures of free-ranging oryx (*Oryx gazella*). Pflügers Arch. 2002;443: 437–445. doi: 10.1007/s004240100704 11810215 10.1007/s004240100704

[41] Ostrowski S, Williams JB, Mésochina P, Sauerwein H. Physiological acclimation of a desert antelope, Arabian oryx (*Oryx leucoryx*), to long-term food and water restriction. J Comp Physiol B. 2006;176: 191–201. doi: 10.1007/s00360-005-0040-0 16283332 10.1007/s00360-005-0040-0

[42] Islam ZM, Wacher T, Boug A, Wronski T. Population development of re-introduced mountain gazelle in the western Empty Quarter (Uruq Bani Ma’arid Protected Area), Saudi Arabia. Global re-introduction perspectives. Glob Re-introduction Perspect. 2011; 180–184.

[43] Mandaville JP. Flora of Eastern Saudi Arabia. London: Kegan Paul International; 1990.

[44] Mandaville JP. Plant life in the Rub’al-Khali (the Empty Quarter), south-central Arabia. Proc R Soc Edinburgh B. 1986;89: 147–157.

[45] El-Ghani MMA. Phenology of ten common plant species in western Saudi Arabia. J Arid Environ. 1997;35: 673–683.

[46] Meigs P. World distribution of arid and semi-arid homoclimates. Rev Res Arid Zo Hydrol. 1953;1: 203–209.

[47] Boyles JG, Smit B, McKechnie AE. A new comparative metric for estimating heterothermy in endotherms. Physiol Biochem Zool. 2011;84: 115–23. doi: 10.1086/656724 20979498 10.1086/656724

[48] McKechnie AE, Chetty K, Lovegrove BG. Phenotypic flexibility in the basal metabolic rate of laughing doves: responses to short-term thermal acclimation. J Exp Biol. 2007;210: 97–106. doi: 10.1242/jeb.02615 17170153 10.1242/jeb.02615

[49] Smit B, McKechnie AE. Avian seasonal metabolic variation in a subtropical desert: basal metabolic rates are lower in winter than in summer. Funct Ecol. 2010;24: 330–339.

[50] Burnham KP, Anderson DR. Model selection and multimodel inference. 2nd ed New York: Springer Verlag; 2002.

[51] Brigham RM, Willis CKR, Geiser F, Mzilikazi N. Baby in the bathwater: Should we abandon the use of body temperature thresholds to quantify expression of torpor? J Therm Biol. Elsevier; 2011;36: 376–379. doi: 10.1016/j.jtherbio.2011.08.001

[52] Boyles JG, Smit B, McKechnie AE. Does use of the torpor cut-off method to analyze variation in body temperature cause more problems than it solves? J Therm Biol. Elsevier; 2011;36: 373–375. doi: 10.1016/j.jtherbio.2011.07.007

[53] Muñoz-Garcia A, Ben-Hamo M, Korine C, Pinshow B, Williams JB. A new thermoregulatory index for heterothermy. Methods Ecol Evol. 2014;5: 141–145. doi: 10.1111/2041-210X.12131

